# Engineering α-amylase levels in wheat grain suggests a highly sophisticated level of carbohydrate regulation during development

**DOI:** 10.1093/jxb/eru299

**Published:** 2014-07-22

**Authors:** Alex Whan, Anne-Sophie Dielen, Jos Mieog, Andrew F. Bowerman, Hannah M. Robinson, Keren Byrne, Michelle Colgrave, Philip J. Larkin, Crispin A. Howitt, Matthew K. Morell, Jean-Philippe Ral

**Affiliations:** ^1^CSIRO Food Futures National Research Flagship, GPO Box 1600, Canberra ACT 2601Australia.; ^2^Research School of Biology, The Australian National University, Canberra, ACT 0200, Australia.; ^3^CSIRO Animal, Food and Health Sciences, St Lucia, Queensland, Australia; ^4^CSIRO Plant Industry, GPO Box 1600, Canberra ACT 2601Australia.

**Keywords:** AMY3, wheat, sucrose, oil, starch, endosperm.

## Abstract

Wheat starch degradation requires the synergistic action of different amylolytic enzymes. Our spatio-temporal study of wheat α-amylases throughout grain development shows that AMY3 is the most abundant isoform compared with the other known α-amylases. Endosperm-specific over-expression of AMY3 resulted in an increase of total α-amylase activity in harvested grains. Unexpectedly, increased activity did not have a significant impact on starch content or composition but led to an increase of soluble carbohydrate (mainly sucrose) in dry grain. In AMY3 overexpression lines (A3OE), germination was slightly delayed and triacylglycerol (TAG) content was increased in the endosperm of mature grain. Despite increased AMY3 transcript and protein content throughout grain development, alterations of α-amylase activity and starch granule degradation were not detected until grain maturation, suggesting a post-translational inhibition of α-amylase activity in the endosperm during the starch filling period. These findings show unexpected effects of a high level of α-amylase on grain development and composition, notably in carbon partitioning and TAG accumulation, and suggest the presence of a hitherto unknown regulatory pathway during grain filling.

## Summary

Endosperm-specific over-expression of wheat α-amylase TaAMY3 during grain development revealed unexpected effects on grain composition, carbon partitioning, fatty acid accumulation, and germination.

## Introduction

Alpha-amylase (EC 3.2.1.1) has been recently described as the “best known amylolytic enzyme” (Janecek *et al.*, 2013). However, the role of individual isoforms of α-amylase in cereal starch degradation remains unclear.

Starch is composed of two distinct fractions: amylopectin (highly branched, 75% of starch dried mass) and amylose (mostly linear, 25% of dried mass). Both are made of α-1,4 glucosidic bond glucose residues branched via α-1,6 glucosidic linkages (Ball and Morell, 2003 for review). Starch is usually classified in two categories: (A) transitory starch, which accumulates in the chloroplasts of leaf cells during the day and is hydrolysed during the night and (B) reserve starch, which accumulates in the amyloplasts of starch storage organ cells, such as cereal endosperm or potato tuber, and is used during germination to fuel the first stages of seedling growth.

Starch degradation requires the orchestrated action of a range of enzymes including glucan water dikinases (GWD, PWD), phosphatases (starch excess 4 (SEX4), like-sex-four (LSF1 and LSF2)) and many amylolytic enzymes (Blennow and Engelsen, 2010; Ritte *et al*., 2006, 2002; Streb *et al.*, 2012). The arsenal of enzymes mobilised to digest starch varies between species and starch type, depending on plant requirements (Lloyd *et al.*, 2005).

In developing plants, degradation of transitory starch reaches completion at the very end of the night period (for review, Graf and Smith, 2011). Recently, Scialdone *et al.* (2013) have established a mathematical model that suggests a very precise control of the energy reserves throughout the non-photosynthetic period. Exo-amylases, such as β-amylases, or debranching enzymes, such as isoamylases or limit dextrinases, are the primary enzymes involved in transitory starch degradation. *Arabidopsis* plants lacking β-amylase (BAM1) showed a severe starch excess phenotype associated with slower development owing to their inability to remobilize starch at night (Fulton *et al.*, 2008).

To provide a steady supply of rapidly metabolised sugars such as glucose and maltose, starch needs to be degraded progressively during the night. Different control mechanisms have been proposed. Light-induced redox control or circadian rhythm are considered to be the primary factors behind regulation of starch degradation (Mikkelsen *et al.*, 2004; Ral *et al.*, 2006). Carbon balance and sugar sensing have recently been described as another potential mechanism controlling starch degradation. Weise *et al.* (2006) reported that starch degradation was strongly controlled by the carbon balance, inhibited when the carbon presence is high and stimulated when the pool of carbon available is low. The work highlighted maltose and glucose as the primary metabolites produced from starch degradation to provide substrates for sucrose biosynthesis; they have been described as major regulators of starch degradation. *Arabidopsis* mutants unable to export maltose from the chloroplast accumulate chloroplastic maltose, which results in a severe starch excess phenotype (Niittylä *et al.*, 2004). High levels of sucrose in *Arabidopsis* leaves have been shown to inhibit starch degradation strongly via another sugar-signalling metabolite, trehalose-6-phosphate (T6P). Although the mechanism underlying the inhibition of starch degradation by T6P remains unclear, leaf starch excess associated with altered starch granule morphology were described in *Arabidopsis* mutants accumulating T6P (Martins *et al.*, 2013).

In non-photosynthetic organs, high levels of reserve starch need to be mobilized very rapidly during germination and early seedling growth. For most cereals, α-amylases are the primary enzymes responsible for starch degradation during germination. Alpha-amylases are endo-hydrolases belonging to the GH13 family (Majzlova *et al.*, 2013). The roles and number of isoforms vary across the plant kingdom. In *Arabidopsis*, three isoforms have been described: AtAMY1, AtAMY2, and AtAMY3 (Yu *et al.*, 2005). AtAMY3 is the only plastidic alpha-amylase, whereas AtAMY1 and AtAMY2 do not have any chloroplast targeting peptide (Stanley *et al.*, 2002). Initially AtAMY3 was thought not to be required for transitory starch metabolism, as mutation of the gene did not affect the starch biosynthetic pathway (Yu *et al.*, 2005). However, when AtAMY3 knock-out was combined with knock-outs of debranching enzyme and/or limit dextrinase deficient lines, the importance of AtAMY3 in transitory starch catabolism was demonstrated. AtAMY3 acts on starch granules producing short branched glucans within the chloroplast stroma. These branched glucans are then rapidly converted into maltose and glucose by debranching enzymes (Streb *et al.*, 2012). AtAMY3 has been demonstrated to be under redox control and potentially involved in redox-regulated, stress-induced starch degradation, particularly in response to osmotic stress or cold shock (Seung *et al.*, 2013).

In barley there are at least four α-amylase categories from HvAMY1 to HvAMY4 (Radchuk *et al.*, 2009), whereas rice contains multiple isoforms from ten separate genes clustered into three subfamilies (OsAMY1, OsAMY2 and OsAMY3, Huang *et al.*, 1992). All subfamilies in barley or rice have been demonstrated to be expressed at different grain developmental stages and in various tissues (Hwang *et al.*, 1999). In wheat, three isoforms of α-amylase have been clearly identified, and a fourth has been suggested based on detection of expression (Barrero *et al.*, 2013). The two major isoforms, TaAMY1 and TaAMY2 have been extensively characterised and isolated based on their isoelectric point (Ainsworth *et al.*, 1985). Wheat lines with abnormal accumulation of these α-amylases have reduced starch viscosity, which is correlated with poor flour quality, most notably in the case of TaAMY2 and pre harvest sprouting (Gubler *et al.*, 2005; Mares and Mrva, 2008).

Although TaAMY1 and TaAMY2 have been well described, very little is known about TaAMY3 in wheat. However, the gene encoding TaAMY3 seems to be expressed throughout the grain development which implies a role in grain maturation (Baulcombe *et al.*, 1987).

Here, we present the spatio-temporal characterization of wheat TaAMY3 and its relative importance compared with the other known α-amylases during kernel development. We also describe the development of endosperm-specific TaAMY3 overexpression wheat lines (A3OE) and the potential consequences of increased TaAMY3 activity on starch structure and granule morphology. We report unexpected effects of high levels of α-amylase level on grain composition, carbon partitioning, and lipid accumulation and finally speculate on the potential role of soluble carbohydrate in the regulation of starch metabolism during grain early development and grain filling.

## Material and methods

### Vector construction and wheat transformation

Nucleotides 1–2397 of a wheat *AMY3* genomic DNA sequence (Genbank Accession **#** X05809) were cloned in the sense orientation into the vector pBx17IRcasNOT (Ral *et al.*, 2012) and placed under the control of the endosperm-specific *Bx17* promoter (Supplementary Fig. S1A, available at *JXB* online). Analysis of the sequence using ChloroP (Emanuelsson *et al.*, 1999) indicated the presence of a plastid targeting signal.

Transformation was carried out using the method of Pellegrineschi *et al.* (2002). Immature embryos were co-bombarded with the AMY3 OE construct and pCMneoSTLS2 (Maas *et al.*, 1997).

### Plant rearing and sampling

Plants were grown in glasshouses at CSIRO Plant Industry, Canberra, Australia, under natural light on a diurnal temperature cycle of 14/20 °C. T0 plants and Chara plants used for wild-type α-amylase characterization were grown in 15cm pots and watered daily. All later stage GM-plants and their negative controls were grown in simulated plot trials as described in Ral *et al.*, (2012), unless described otherwise. Simulated plot trials were watered automatically at a rate equivalent of 10mm of water every 3 d.

Developing grains samples (dissected where appropriate) and other tissue samples were frozen on dry ice and stored at –80 °C until time of analysis.

For phenotypic characterization, T3 plants were grown in replicated blocks of nine 10cm pots. Each genotype had three or four blocks in the trial, resulting in 27–36 replicates per line. At maturity, plant material was cut at soil height and weighed. Spikes were removed and weighed, after which they were threshed in a single head thresher. Total grain yield was recorded, as well as thousand grain weight.

### Germination

Coleorhiza and root emergence was measured in light and dark conditions for two A3OE lines, A4 and A10. Seeds were obtained from T3 plants and studied in four replicates of 20 grains placed on two layers of Whatman #1 filter paper in a petri dish. Five ml of distilled water was added and the plates sealed and incubated at 20 °C. Plates were placed under constant light conditions and received 126 µm⁻^2^ s⁻^1^ of photons.

### α*-Amylase assay*

Alpha-amylase activity was determined in 10mg wholemeal samples and 2–6 ground developing grain samples. The CERALPHA kit (Megazyme International Ireland Ltd) was used, with the manufacturer’s protocol adapted for 96-well format and with appropriate dilutions. Data was expressed in CU (ceralpha unit) per g flour or µg of protein as determined by Bradford assays (Bradford, 1976) on the CERALPHA extracts.

### Western-blot analysis

Proteins were extracted with the CERAPHA kits and quantified as above. Twenty µg of protein from each sample was mixed 3:1 with 3x loading buffer (1M Tris-HCl pH 6.8, 20% SDS, 100% glycerol, β-mercaptoethanol and bromophenol blue) and boiled for 5min. Samples were loaded onto NuSep Tris-HEPES NH 12% precast gels and run at 110V for approximately 60min. Proteins were transferred onto a nitrocellulose membrane and probed using polyclonal antibody raised against selected wheat peptides showing strong distinctive antigenic capacity against TaAMY1, TaAMY2, and TaAMY3 (Genscript, USA). The antigen–antibody complexes were visualized by chemiluminescence (Sigma-Aldrich). The three polyclonal antibodies showed similar affinity for the same concentration of their respective peptides at the used dilution (1/2000).

### Protein extraction and digestion

Triplicate samples from wholemeal flour from A4, A10, and A17 positive and isogenic negative control lines were prepared for α-amylase protein extraction. Milled wholemeal flour (50mg) was dissolved in 500 µl of Ceralpha:α-amylase reagent (50mM sodium malate, 50mM sodium chloride, 2mM calcium chloride, pH 5.4), vortexed briefly, and incubated at room temperature for 20min with agitation. This mixture was then heated (40 °C) and shaken in a thermal mixer for 30min. The samples were centrifuged at room temperature (17 000*g*, 10min). The supernatant was reduced by the addition of 1M dithiothreitol (DTT) at a ratio of 1:10 to reach a 100mM DTT final concentration, followed by 60min incubation (40 °C, 14,000rpm).

Protein extracts (100 µl) were applied to a 10kDa molecular weight cut-off filter (Millipore, Australia), washed with two 200 µl volumes of 8M urea, 100mM Tris-HCl (pH 8.5) with centrifugation at 17 000*g* for 10min. For cysteine alkylation, 100mM iodoacetamide in 8M urea, 100mM Tris-HCl was added (100 µl) and incubated at room temperature in the dark for 30min. The filters were centrifuged at 14 000rpm for 10min to remove excess iodoacetamide and washed with two 100 µl volumes of 8M urea, 100mM Tris-HCl. The buffer was exchanged to prepare the proteins for digestion using 50mM ammonium bicarbonate (pH 8.5) by two consecutive wash/centrifugation steps. The digestion enzyme (trypsin) (Promega, Madison, WI, USA) was added (100 µl, 55 µg ml^–1^) in 50mM ammonium bicarbonate and incubated overnight at 37 °C. The 10kDa filters were transferred to fresh centrifuge tubes and the filtrates (digested peptides) were collected following centrifugation at 14 000rpm for 10min. The filters were washed twice with 100 µl of 50mM ammonium bicarbonate and the filtrates were combined and lyophilized. The tryptic peptides were resuspended in 50 µl of 1% (v/v) formic acid.

### Protein identification

Samples (5 µl) were chromatographically separated on a Shimadzu nano HPLC system (Shimadzu Scientific, Rydalmere, Australia) using a Agilent Zorbax 300SB-C18 300 Å, column (150mm × 100 µm) with a particle size of 3.5 µm using a linear gradient (flow rate of 500 nl min^–1^) from 2–40% (v/v) solvent B over 30min where solvent A was 0.1% (v/v) formic acid and solvent B was 0.1% (v/v) formic acid in 90% acetonitrile. The eluate was directed into the nanoelectrospray ionization source of the TripleTOF™ 5600 system (AB/Sciex, Foster City, USA). Data were acquired in information-dependent acquisition (IDA) mode. The IDA method consisted of a high-resolution TOF-MS survey scan followed by 20 MS/MS in a second with a maximum accumulation time of 50ms. First-stage MS analysis was performed in positive ion mode over the mass range *m/z* 300–1800 with a 0.5 s accumulation time. The ionspray voltage was set to 2200V, the curtain gas was set to 25, the nebuliser gas to 20 and the heated interface was set to 160 °C. Tandem mass spectra were acquired on precursor ions that exceeded 120 cps with charge state 2–5. Spectra were acquired over the mass range *m/z* 80–1800 using rolling collision energy (CE) for optimum peptide fragmentation. Precursor ion masses were excluded for 8 s after two occurrences.

ProteinPilot^TM^ 4.1.46 software (Applied Biosystems) with the Paragon Algorithm was used for the identification of proteins. Tandem mass spectrometry data was searched against *in silico* tryptic digests of Magnoliophyta proteins of the Uniprot database (version 2013/06; 2,804,812 sequences) or against a custom database comprising the three expected α-amylase protein isoforms. All search parameters were defined as iodoacetamide modified with cysteine alkylation, with trypsin as the digestion enzyme. Modifications were set to the “generic workup” and “biological” modification sets provided with this software package, which consisted of 126 possible modifications; for example, acetylation, methylation, and phosphorylation. The generic workup modifications set contains 51 potential modifications that may occur as a result of sample handling; for example, oxidation, dehydration, and deamidation. Peptides with missed cleavages were identified, but were not included in subsequent MRM analysis.

### Multiple reaction monitoring (MRM) assay for α-amylase

MRM transitions were determined for each peptide where the precursor ion (Q1) *m/z* was based on the size and expected charge and the fragment ion (Q3) *m/z* values were predicted using known fragmentation patterns and/or the data collected in the characterization workflows. In the preliminary analyses, up to 10 peptides per amylase isoform were selected with three MRM transitions per peptide. The final MRM method consisted of five peptides for AMY3 with three MRM transitions per peptide, wherein the most intense MRM transition was used as a quantifier and the second and third MRM transitions were used as qualifiers. Reduced and alkylated tryptic peptides were analysed on an AB/Sciex 6500 QTRAP mass spectrometer (AB/Sciex, Framingham, MA, USA) equipped with a TurboV ionization source operated in positive ion mode. Samples (5 µl) were chromatographically separated on a Shimadzu Nexera UHPLC (Shimadzu) using a Phenomenex Kinetex C18 (2.1mm × 10cm) column with a linear gradient of 5–45% (v/v) acetonitrile (ACN) over 10min with a flow rate of 400 µl min^–1^. The eluent from the HPLC was directly coupled to the mass spectrometer. Data were acquired and processed using Analyst 1.6 software^TM^. Quantification of the α-amylase tryptic peptides was achieved using MRM experiments using a 5ms dwell time. The scan speed was set to 1000Da s^–1^ and peptides were fragmented in the collision cell with nitrogen gas using collision energy dependent on the size and charge of the precursor ion. The MS parameters were: ionspray voltage (IS) 5500V, curtain gas 35, GS1 40, GS2 50, source temperature 500 °C, declustering potential (DP) set to 70 and entrance potential (EP) set to 10. Peaks were integrated using MultiQuant v3.0 (AB/Sciex) and graphed using Excel and or GraphPad Prism v6.

### Real-time PCR

Construct copy numbers were determined as described in Mieog *et al.* (2013). For gene transcript levels, vegetative plant material and seeds were obtained from T3 plants and put in masterblock wells together with a 5mm stainless steel ball and a few milligrams of sand on dry ice. The blocks were capped, put at –80 °C for at least 30min before being shaken in a Retch MM300 ball mill for 30 s at 25/s frequency. Next, 500 µl of heated extraction buffer (Schneiderbauer *et al.*, 1991) was added, the block recapped and the samples incubated for 15min at 65 °C with regular mixing. After centrifugation (5000g, 1 minute) the supernatants of grain samples were extracted with 300 µl chloroform before 300 µl of 95% (v/v) ethanol was added. For other tissues, 300 µl 95% (v/v) ethanol was added directly after the centrifugation step. The samples were then loaded in nucleospin RNA columns (Macherey-Nagel) and RNA was purified following the manufacturer’s protocol.

Following RNA isolation, samples were DNAse I treated (Sigma) and diluted to 5–10ng µl^–1^. Real-time PCR reactions were run as described previously (Mieog *et al.*, 2013) with the following modifications. Reactions consisted of: 10 µl SensiFAST SYBR & Fluorescein one-step, 4.4 µl primer mix (1.6 µM of forward and reverse primer), 0.2 µl reverse transcriptase, 0.4 µl RNAse inhibiter, 5 µl RNA template. Before the actual real-time PCR run, a single step of 10min at 45 °C was inserted into the protocol. Reactions were run in duplicates.

For normalization, existing primers were used that targeted the actin gene (Ji *et al.*, 2011); TaActinFP: TCAGCCGAGCGGGAAATTGT, TaActinRP: CCTCTCTGCGCCAATCGT. New primers were developed for *TaAmy3* following the procedures described in Mieog *et al.* (2013); TaAmy3FP: TTCTTTTCCAGGGGTTTAATTGGG, TaAmy3RP: CTCCACCTTCCCTTGCATGA.

### Microscopy

Seeds from T3 plants were sampled from line A10 and its isogenic control during the starch filling phase (approx. Z73 on the Zadoks scale) and during the desiccation phase (approx. Z80).

For confocal microscopy, 2 µm sections of fixed, embedded seeds were sectioned on a microtome. Sections were stained with iodine solution for 5min, rinsed with water for 5min and observed under bright field, using a Leica SP2 confocal laser scanning microscope.

For scanning electron microscopy, starch granules extracted following the method described in Ral *et al.* (2012) were sputter coated with ~20nm gold using an Emitech K5000X sputter coater. Samples were then visualized in a Zeiss EVO LS 15 extended pressure-scanning electron microscope, in extended pressure mode (10 Pa chamber pressure, 20 °C Peltier stage temperatures, 10–15kV accelerating voltage). Images were processed (contrast adjusted) using Photoshop CS2.

### Carbohydrate measurement

Frozen developing grains and dry grains from T3 plants were ground with a hammer mill. Three times 50mg aliquots were extracted three times in 400 µl boiling 80% (v/v) ethanol each. Water soluble carbohydrates were measured in the supernatants pooled per aliquot. Total soluble sugars, total sucrose, free fructose, and free glucose were measured as described by (Campbell *et al.*, 1999). Maltose and total starch were measured using the Maltose and Total Starch assay kits from Megazyme, respectively. All spectrophotometric measurements were performed using a Thermo Multiscan Spectrum plate reader after appropriate dilutions.

### Triacyl glycerol (TAG) measurement

TAG from embryos and endosperms of dry grains from T3 plants were extracted and analysed following the method described in Vanhercke *et al.* (2013). Each biological replicate contained tissues from five grains. Three technical replicates of three biological replicates were done per lines analysed.

### Statistical analysis

All statistical analyses were performed with the mixed modelling software ASReml-R (Butler *et al.*, 2007), which has been developed as an R package (R Core Team, 2013). For carbohydrate measurements on mature grains (total starch, total soluble sugars, total sucrose, free fructose, free glucose, and maltose) a model was fitted treating presence of the construct and the genetic background as fixed effects. Where observations were made on developing grains, and assays were extended over multiple plates, (α-amylase activity and sucrose concentration), non-genetic variation owing to experimental processes, such as day of extraction and plate location, were fitted as random effects. For plant growth and yield traits, the same base model was used, and position on the glasshouse bench was fitted as a random effect. Predicted means and standard errors were derived from the ASReml-R predict method. Analysis of germination rates were performed using modified Kaplan-Meir plots and Survival analysis using the “survival” package in R (http://cran.r-project.org/web/packages/survival/index.html), indicating the likelihood of germination at a given time point. Differences in germination rates are compared using a log-rank test, and hazard ratios are produced using Cox Proportional hazards.

## Results

### Wild-type α-amylase activity and localization

**Fig. 1.**
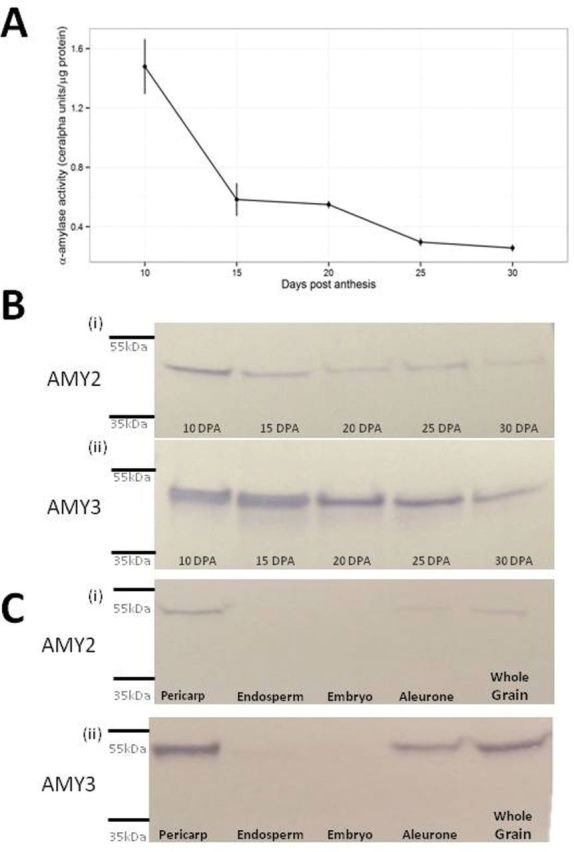
Total α-amylase activity and tissue localization in developing grain of wild-type Chara. (A) Total α-amylase activity measured against extractable protein (Unit per µg protein) in developing grain. Grain collected at 10, 15, 20, 25, and 30 DPA. (B) Western blots on proteins samples from extraction performed in (A). Equal amount of extracted proteins (20mg) were loaded from each time point and probed with polyclonal antibodies raised against α-amylase2 (TaAMY2, panel B (i) and α-amylase3 (TaAMY3, panel B (ii)). (C) Tissue-specific α-amylase expression from 12 dissected developing grains at 20 DPA: C (i) TaAMY2, C (ii) TaAMY3).

Total α-amylase activity was measured in untransformed wheat seeds (*Triticum aestivum* L. var. Chara), sampled at 10, 15, 20, 25 and, 30 days post anthesis (DPA). Total activity was highest at 10 DPA, decreased rapidly between 10 DPA and 15 DPA, and declined further by 30 DPA (Fig. 1A). Western blots from the same timepoints showed a similar pattern of decreasing protein content during development, with TaAMY3 being more abundant than TaAMY2 signal at all time points (Fig. 1B).

TaAMY2 and TaAMY3 had similar localization patterns at 20 DPA, with the highest level in pericarp, followed by aleurone (Fig. 1C). Protein was not detected in either endosperm or embryo.

TaAMY1 protein was not detected in any tissue at any timepoints (data not shown).

### Development of TaAmy3 overexpression lines

To investigate the role of TaAMY3 in the grain we developed transgenic lines over-expressing the full *TaAmy3* gDNA. The *Bx17* glutenin promoter (Ral *et al.*, 2012) was chosen to specifically target the endosperm and to affect the TaAMY3 content from the early stage of seed development (see Supplementary Fig. S1A, available at *JXB* online).

Fifteen *TaAmy3* over-expressing (A3OE) geneticin resistant wheat T0 plants were selected by PCR using construct-specific primers after biolistic-mediated transformation of immature embryos. Using real-time PCR, we estimated the number of insertions for each event at the T1 generation, which ranged from 1–9.

**Fig. 2.**
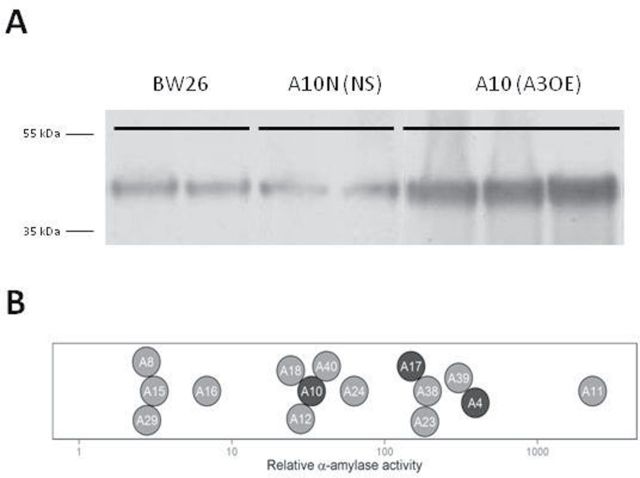
Effectiveness of TaAMY3 over-expression construct. (A) Representative Western blot for TaAMY3 protein content in A3OE lines. Total proteins were extracted from 25 DPA grains from the parent (bw26), a positive line (A10), and its isogenic negative segregant (A10N) at the T0 plant stage. Western-blot was performed using a TaAMY3-specific antibody. (B) 1D striplot of the relative α-amylase activity compared with parental line BW26 for best T1 grain per events (triplicate measurements). Further characterizations were performed on representative selected lines (dark grey dots) (see Supplementary Table S1, available at *JXB* online).

Relative protein quantification on mature T1 grains compared with parental line BW26 using a TaAMY3-specific antibody showed a wide range of TaAMY3 protein, with a 1.2–53.1-fold increase (Fig. 2A and Supplementary Table S1, available at *JXB* online). Among the transformed lines, the level of amylase activity increase ranged from 2.1 to over 2000-fold compared with the untransformed control (Fig. 2B and Supplementary Table S1, available at *JXB* online).

Owing to the large diversity in copy number and total α-amylase activity, a representative panel of three homozygous lines from independent events, labelled A4 (single insert), A10 (single insert) and A17 (double insert), together with their isogenic negative segregants (A4N, A10N, and A17N respectively) were chosen for further characterization. Those three independent events showed an increase in amylase activity of 392-, 33-, and 149-fold, respectively, compared with the parent line BW26 (Fig. 2B and Supplementary Table S1, available at *JXB* online).

### TaAmy3 transcript

**Fig. 3.**
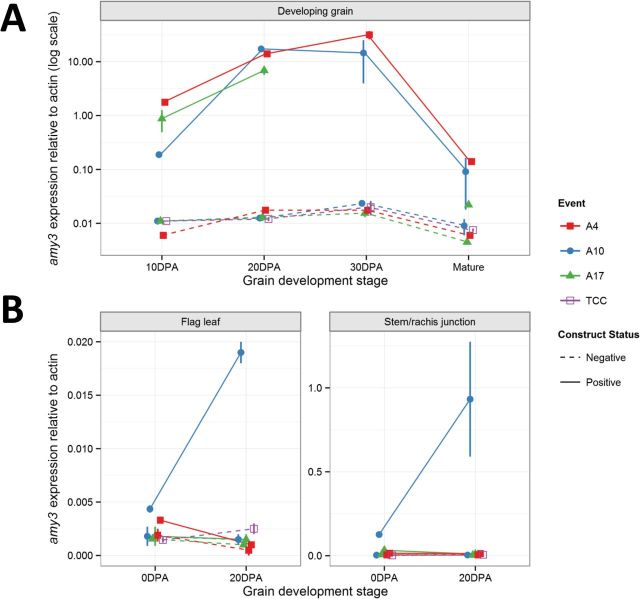
TaAMY3 relative transcript level and tissue specificity during grain development. TaAMY3 transcript level (relative to actin) during seed development in whole grain (A), flag leaf (B), and stem/rachis junction (C). Positive (A) lines were compared with their negative isogenic controls (AN) and Bobwhite 26 tissue culture control (TCC). Notice the logarithmic Y-scale.

Using real-time PCR, we measured the level of the *TaAmy3* transcript (relative to actin) during grain development. In the transgenic lines, the level of *TaAmy3* relative expression increased from 10 to 20 DPA, remained about this level till 30 DPA and was decreased by maturity (Fig. 3A). Relative transcript in isogenic negative segregants increased from 10 to 20 DPA, and remained steady until grain maturity. At all time points transcript was increased in A3OE lines as would be expected from the *Bx17* promoter employed. To check for unintended tissue expression, *TaAmy3* transcript was measured in flag leaf and the stem/rachis junction at 0 and 20 DPA. In one of three stable A3OE lines (A10), a significant increase in *TaAmy3* transcripts was observed in the stem and flag leaf (Fig. 3B) at 0 and, most notably, 20 DPA. However, no increase in amylase activity could be detected in these tissues (Supplementary Fig. S1B, available at *JXB* online).

### A3OE grain composition

**Fig. 4.**
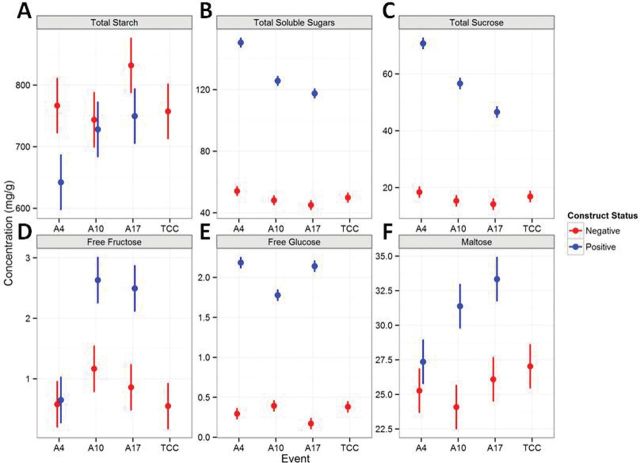
Carbohydrate composition of A3OE lines in mature grain. Six carbohydrate measurements on the same extracts from dry T4 grains. Positive lines (blue) compared with isogenic and tissue culture controls (red). Concentrations are expressed in mg.g dry weight^–1^.

No significant differences were detected in mature grain starch content between the A3OE lines, their isogenic negative segregants and tissue culture control (TCC, Fig. 4A). All three A3OE lines had 2.6—2.8-fold higher levels of total soluble carbohydrates in the mature grain compared with their isogenic controls (*P*<1×10^–10^, Fig. 4B). Each of the soluble sugars analysed were significantly increased in positive lines (*P*<0.001). The magnitude of the increase was similar to that of total soluble carbohydrate with the notable exception of maltose. For instance, fructose increased 2.5-fold on average (Fig. 4D) and the major component of the soluble sugars, sucrose, increased 3-fold (Fig. 4C). Glucose showed the highest increase (6-fold, Fig. 4E) but remained a minor component of the total soluble carbohydrate. Maltose was increased by approximately 1.2-fold (Fig. 4F).

**Fig. 5.**
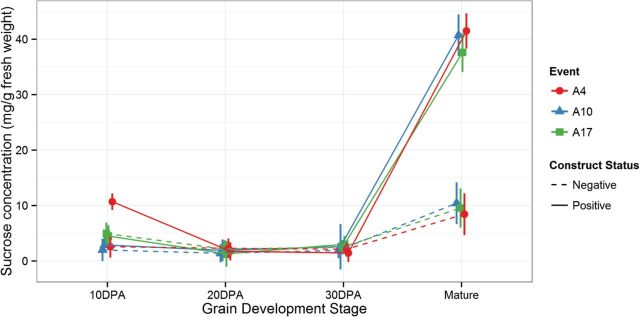
Sucrose concentration during grain development. Sucrose from developing T4 grains. Positive lines (blue) compared with isogenic and tissue culture controls (red). Concentrations are expressed in mg.g fresh weight–1.

Although sucrose is not the primary product of starch degradation by α-amylase, it was the main constituent of the soluble carbohydrates. In the developing grain, total sucrose levels remained almost identical in the A3OE lines compared with their isogenic negative segregants until 30 DPA. After 30 DPA, the sucrose increased 4-fold compared with the negative segregants (Fig. 5).

**Fig. 6.**
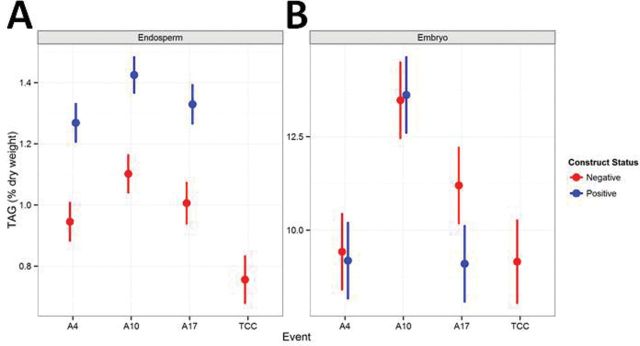
TAG concentration during grain development. Triacyl glycerol (TAG) concentration in endosperm and embryo of A3EO lines. Positive lines (blue) compared with isogenic and tissue culture controls (red). Concentrations are expressed in mg.g dry weight^–1^.

Triacylglycerol (TAG) was increased significantly in the endosperm of mature grain (*P*=0.0086). A3OE lines showed a 31–42% increase in TAG in the endosperm compared with their isogenic negative segregants (Fig. 6A). However, there was no significant change in TAG content in the embryo (Fig. 6B).

### Engineered AMY3 profiling during grain development

The presence of high levels of soluble carbohydrates, sucrose in particular, within the mature grain of the A3OE lines suggested an impact of TaAMY3 on the starch granules. However, the absence of a significant reduction in the total starch content of the mature grain despite very large increases on TaAMY3 activity suggests some limitations of TaAMY3 in hydrolysing the starch granule efficiently.

**Fig. 7.**
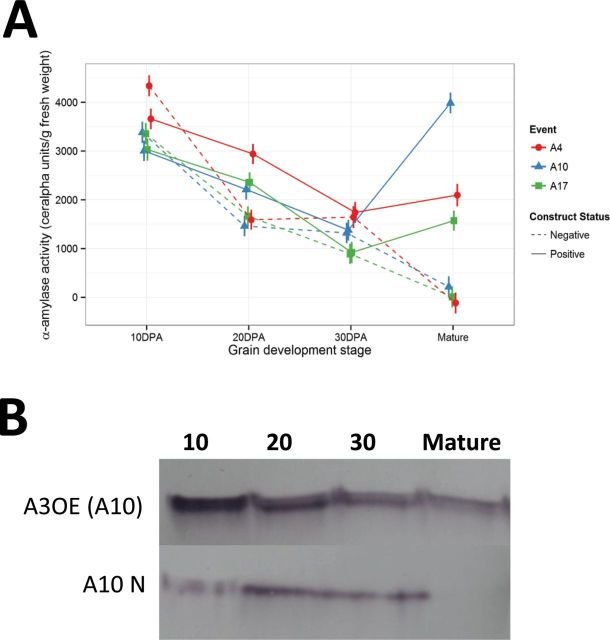
Total α-amylase activity and TaAMY3 protein content. (A) Total α-amylase activity in A3OE and negative isogenic controls during grain development (T4 grains). (B) Representative Western blot of A3OE line A10 and its negative isogenic control A10N at 30 DPA in T4 grains using specific anti-TaAMY3 antibody.

**Fig. 8.**
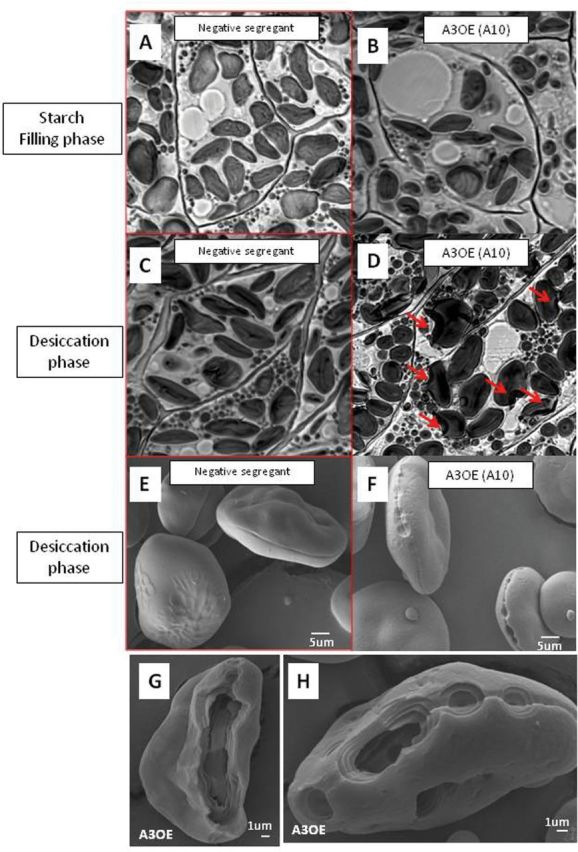
Impact of TaAMY3 over-expression on starch granules during seed development. (A–D) Evolution of starch granule morphology during seed development as visualized by confocal microscopy. Developing grains from a negative isogenic control (A and C) and an AOE line (B and D) were collected during the starch filling process (A and B) and at the beginning of the desiccation phase (C and D). Degradation signs are indicated by red arrows. (E–H) Impact of α-amylase3 (TaAMY3) over-expression on starch granules extracted from dry grains as visualized with SEM. (E) A10 negative isogenic control. (F–H) A10 A3OE line.

Therefore we assessed the total α-amylase activity (Fig. 7A), the presence of the TaAMY3 protein (Fig. 7B), and starch granule morphology during grain development (Fig. 8). Isogenic negative segregants showed a similar pattern to the wild-type wheat (Fig. 1A) characterized by decreasing total α-amylase activity during grain development to nearly zero at maturity. The positive lines showed a similar pattern of decreasing total α-amylase activity up to 30 DPA, but then increased during maturation (Fig. 7A). In negative segregants, TaAMY3 protein levels seemed to increase from 10 to 20 DPA, remain stable to 30 DPA and decrease to very low levels at maturity. In contrast, protein levels were highest in A3OE lines at 10 DPA, and decreased during development. Despite the steady decrease during development, protein levels were higher in A3OE lines (Fig. 7B). Western-blot profiling showed the presence of two forms of TaAMY3 (Supplementary Fig. S4, available at *JXB* online) separated by a few kDa. In the A3OE line, the larger form was mainly present before and after the endosperm starch filling process and the smaller form was the dominant form detected during endosperm starch filling. Relative quantification measured with multiple reaction monitoring (MRM) mass spectrometry showed that peptides derived from the TaAMY3 protein were significantly higher (≥45-fold increase) in the A3OE lines than the negative segregants (Supplementary Fig. S3, available at *JXB* online). Peptides targeted for TaAMY1 and TaAMY2 were below the detection limit in all samples tested. This result confirmed the engineered TaAMY3 to be responsible for the α-amylase activity increase detected in mature grain.

Confocal microscopy showed that during grain fill, starch granule morphology was normal (Fig. 8A and B). However, during maturation, and only in the A3OE lines, the surfaces of starch granules were affected, and partial digestion was visible (Fig. 8C, D). Scanning electronic microscopy (SEM) of starch granules from maturing A3OE lines showed the initiation of the digestion (Fig. 8E, F). Furthermore, distinct and localized digestion was visible on the equatorial grooves characteristic of the wheat starch granule (Fig. 8G, H).

Consistent elevated *TaAmy3* transcript and increased AMY3 protein in the A3OE lines without a detectable change in α-amylase activity indicates potential inhibition of α-amylase activity during the starch filling period of developing grain at either the translational or post-translational level.

### Impact on growth and yield

No large phenotypic differences were detected during plant development between A3OE plants and isogenic negative controls (Supplementary Fig. S2, available at *JXB* online). Of the measured plant growth and grain yield characteristics (total biomass, spike biomass, vegetative biomass, total seed weight, thousand grain weight and total spikes) there were no significant effects due to presence of the overexpression construct, except for a weakly significant (*P*=0.03) decrease in thousand grain weight for positive lines.

**Fig. 9.**
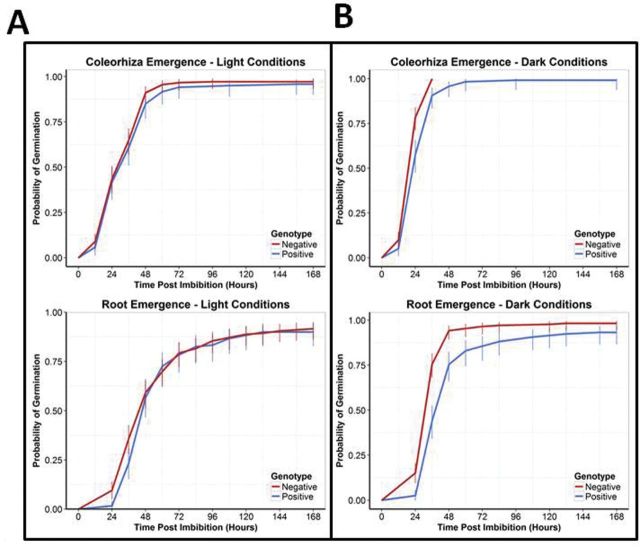
Probability of coleorhiza and root emergence in both constant light and dark conditions. Probability of coleorhizae (top) and root (bottom) emergence during germination under both light (left) and dark (right) conditions. No significant differences could be seen for either root or coleorhizae emergence in light conditions, but a significant delay (*P*<0.001) is found for both under dark conditions, where full germination potential is not reached. Cox proportional hazard indicates that there is a hazard of –0.56 and –0.67 for coleorhizae and roots, respectively, from A3EO lines under dark conditions.

There was a significant delay in coleorhiza (*P*=4.03×10^–6^) and root (*P*=2.34×10^–8^) emergence under dark conditions; however no significant difference was found when grains were germinated in light (Fig. 9).

## Discussion

The importance of α-amylase during grain development has been previously suggested in cereal (Barrero *et al.*, 2013; Hwang *et al.*, 1999). However, results from this project have identified the relative importance of the specific isoform TaAMY3 and suggested the presence of an apparent control over α-amylase activity in wheat during the grain-filling process.

### TaAMY3 is the prevalent α-amylase isoform in developing wheat grains

Grain development can be divided into three parts: cell division and endosperm differentiation from anthesis to 15 DPA; grain filling, where starch is accumulated in the endosperm, from 15 to 35 DPA; and grain desiccation and ripening from 35 DPA until dry (for review Evers, 1970). The length of each period is highly dependent on environmental conditions such as temperature, day length, or moisture (Emes *et al.*, 2003). The greater protein content of TaAMY3 compared with TaAMY2 at all sampled time points from 10–30 DPA and the absence of detectable level of TaAMY1 indicates that TaAMY3 is the most abundant α-amylase isoform in wild-type developing wheat grains. The localization of protein to the pericarp and aleurone also suggests a well-controlled, tissue-specific regulation. In barley, it has been proposed that the pericarp of developing grains acts as a major short-term starch storage tissue, ensuring sink strength of the grain (Radchuk *et al.*, 2009). Noticeably, one pathway involving α-amylases occurs in tissues such as the pericarp and nucellus undergoing programmed cell death. Once accumulated during cell differentiation, starch is degraded during the seed-filling period, ensuring relocation of sugars (mainly sucrose) from the pericarp into the starchy endosperm.

Our goal was to investigate the effects of altering the controlled distribution of TaAMY3 during grain development and its implications for carbohydrate supply during germination. Therefore, we chose to use an endosperm-specific promoter, as no α-amylase isoform was found in that tissue in wild-type grain at 20 DPA (Fig. 1). Previous work has shown that a thermophilic α-amylase only expressed in non-endosperm tissues, spatially separated from starch, did not show any alterations in germination or development (Lanahan *et al.*, 2006), and was only capable of hydrolysing starch after the tissue was ground.

Despite the over-expression construct being controlled by the endosperm specific promoter *Bx17* (Ral *et al.*, 2012), we have identified some ectopic non-endosperm transcript in line A10. However, the levels detected were low compared with those in the endosperm, and diminished with distance from the grain. There was also no detection of increased α-amylase activity outside of the endosperm, and no overall plant morphology or yield change in A10 line compared with the other A3OE lines (Supplementary Fig. S1, available at *JXB* online). Nevertheless, presence of elevated level of *TaAmy3* transcript outside of the grain in one of the A lines is intriguing and needs to be investigated further.

### High endosperm α-amylase activity does not significantly affect starch content or grain yield.

One of the most surprising results was the minimal effect on either the overall grain weight or the starch content of the A3OE grain. Although traces of degradation were clearly visible on the starch granules from mature grains (Fig. 8), the efficiency of degradation was not as high as would be expected from such an increase of α-amylase. This apparent contradiction may be explained via several different mechanisms.

Our first hypothesis was that the absence of digestion, at least until the end of the starch-filling process, could be an indication of different localization between the engineered TaAMY3 enzyme and its substrates. During grain fill, starch in cereal grain is produced in amyloplasts. During maturation, the grain triggers a rapid net water loss and the membrane of the desiccated amyloplasts degrades (Pepler *et al.*, 2006). If TaAMY3 is located outside of the plastid, the degradation of the granule could only occur when the lipid membrane surrounding the amyloplast breaks down.

However, there are good reasons to believe that the over-expressed protein is in amyloplasts during development. Firstly, the *TaAmy3* sequence used for the construct shows the presence of a plastid targeting signal increasing the likelihood of an amyloplast localization of the engineered TaAMY3 protein during the grain development. The presence of the smaller form of TaAMY3 protein is consistent with cleavage of the transit peptide during passage across the amyloplast membrane (Lao *et al.*, 1999) and therefore an amyloplastic localization of the TaAMY3 protein during the starch-filling process. Sequence analysis using peptide prediction software ChloroP (Emanuelsson *et al.*, 1999) confirmed the size of the TaAMY3 transit peptide to be near 3kDa. The presence in mature grain of the TaAMY3 precursor form is probably due to a loss of amyloplast integrity, which would allow the non-cleaved protein access to starch granules. Secondly, the A3OE profiling during grain development showed an absence of elevated total α-amylase activity in crude extract from total grain despite an increase in TaAMY3 protein. This suggests that the lack of starch granule digestion during grain fill is due to α-amylase inactivation rather than physical separation. Purified amyloplasts during grain development would represent the ideal material to assess the localization of the A3OE protein within the endosperm and to identify the mechanism controlling amylase activity.

Another possible explanation is that the affinity for the TaAMY3 for the starch granule is relatively low. The specific location of the hydrolysis on the groove surrounding the granule suggests some limited entry point during the starch granule degradation. Amylases in general and α-amylases in particular show different affinity, mode of action, or efficiency depending on the presence, the amount, and the type of carbohydrate binding domain (CBM) or specific domain called the “Sugar Tong” (Janecek *et al.*, 2013). For example, the AtAMY3 shows a unique and characteristic double CBM45, whereas the other known AMYs display a single CBM20 (Glaring *et al.*, 2011).

The final explanation for the lack of a major difference in the overall starch content would be the inhibition of all α-amylase activity including the over-expressed TaAMY3 during the grain filling process of the transgenic lines. Despite elevated *TaAmy3* transcript and higher protein content, the level of α-amylase activity in the A3OE lines was reduced to wild-type level during the grain-filling process, indicating translational and/or more likely post-translational regulation of activity. The regulation was reduced or removed during maturation, as α-amylase activity was increased in A3OE lines during that phase, despite protein levels being apparently reduced. Between the removal of inhibition and complete desiccation of the grain, there seems to be sufficient time to allow the partial degradation of starch granules seen in the SEM images (Fig. 8), and increase in the soluble carbohydrate pool without significantly impacting overall starch content.

### Over-expression of TaAMY3 suggests post-transcriptional regulation during wheat grain filling

The absence of transcript repression in our AMY3 A3OE and elevated protein in A3OE lines suggests the presence of inhibitors or post-transcriptional inhibition affecting the TaAMY3 during the carbohydrate accumulation process.

Alpha-amylase inhibitors have been described for many years and are exclusively located within the endosperm in wheat (Buonocore *et al.*, 1977). However, they are not active against the endogenous α-amylase like the over-expressed TaAMY3 used in our transgenics.

Alpha-amylases have been shown to be potentially phosphorylated or redox-activated (Glaring *et al.*, 2012; Heazlewood *et al.*, 2008) in *Arabidopsis*. Seung *et al.* (2013) demonstrated light activation of AtAmy3 suggesting a major role of α-amylase in a stress-response mechanism. In addition, RNAi-mediated inactivation of thioredoxin showed clear reduction of starch-related enzyme activities, including α-amylase and β-amylase (Guo *et al.*, 2011). Light-induced activation in a non-photosynthetic tissue seems very unlikely. However, a potential effect of respiration change affecting redox statute of starch-related enzymes cannot be discounted. Respiratory changes in relation to endosperm starch synthesis have been described (Emes *et al.*, 2003). This study suggested the presence of respiratory metabolism partitioning between protein and starch synthesis during endosperm development.

Recent work has highlighted the involvement of sucrose, via T6P, in a negative feedback loop of starch degradation in *Arabidopsis* (Martins *et al.*, 2013). The model suggests that high levels of sucrose would increase T6P accumulation, which would then trigger a feedback mechanism that switches from starch catabolism to biosynthesis.

High levels of sucrose in the mature grain could potentially trigger similar inhibitory effects during the early stage of the grain germination, explaining the observed delay in emergence of A3OE roots and coleorhizae. Seed-specific GWD RNAi in wheat showed a delay in germination associated with a significant increase of α-amylase (Ral *et al.*, 2012). The similarity between the phenotype of the two transgenics suggests that delay could be due to the α-amylase increase, rather than a starch phosphate content alteration. Further characterization needs to be undertaken to identify the regulatory mechanism involved.

### Maintaining energy homeostasis at all costs

The profiling of soluble carbohydrates present in the mature grain of A3OE lines showed a slight but significant increase in maltose along with a high level of glucose and sucrose. Although the excess of maltose and glucose are understandable as they are products of starch degradation by α-amylase (Yu *et al.*, 2005), the very high level of sucrose in the mature grain is intriguing.

The sucrose biosynthesis pathway has been well described in transitory starch metabolism (for review, Koch, 2004). In aerial tissues, photosynthetic energy is initially captured as sucrose and excess is stored via starch accumulation. Starch is then mobilized at night, releasing maltose and glucose that can be exported and converted into sucrose, ensuring readily available energy for plant processes. Sucrose is also the major transport disaccharide and its role in regulation of plant development and physiology has been well described (for review Wind *et al.*, 2010).

In the wheat grain, sucrose is mostly present at the early stage of grain development. During the grain-filling process, the conversion of sucrose from sink tissue into the precursor of starch synthesis, ADP–glucose (ADP–glc), ensures starch accumulation in the endosperm (Dale and Housley, 1986). High levels of sucrose in the A3OE mature grain suggest an additional mechanism converting the α-amylase degradation products (maltose then glucose) into sucrose during grain development. The role of sucrose in a regulatory mechanism for growing tissues and cell differentiation has been described in non-photosynthetic tissues (Martinez-Barajas *et al.*, 2011).

In potato, high levels of sucrose have been shown to redox-activate the ADP–glucose pyrophosphorylase (AGPase) responsible for providing the unique precursor of starch biosynthesis, ADP–glc (Tiessen *et al.*, 2002). In addition, sucrose, acting via T6P, has been identified as an inhibitor of the main regulator of the stress response and sugar signalling SNF1-related kinase 1 (SnRK1, Zhang *et al.*, 2009). In rice, α-amylase 3 has been characterised as a component of a sugar response complex including SnrK1 and a transcription factor MYBS1 (Lu *et al.*, 2007). However, the mechanism through which sucrose is sensed and the components directly inhibiting or enabling starch degradation remain unknown.

The presence of an elevated level of sucrose while not being the primary product of starch degradation by α-amylase suggests the presence in developing grain of a similar alternative pathway sensing and recycling degradation products into usable sugar ensuring energy homeostasis during grain development.

### Lipids as an alternative storage form for starch degradation product?

Despite having a much higher concentration in TAG compared with the endosperm (Morrison, 1977), A3OE embryos didn’t show any alteration of TAG concentration compared with the controls. However, TAG was increased in the mature endosperm of the A3OE lines where starch had been partially degraded at the end of grain development.

Interactions between starch and oil accumulation pathways have been shown in a range of experimental contexts. Starchless mutants of the unicellular *Chlamydomonas reinhardtii* produced over 35% more TAG than the wild type (Work *et al.*, 2010). *Arabidopsis* AGPase RNAi and *Wrinkled1* overexpression double transgenics have also been described in which a significant decrease in starch content was associated with an elevated oil level. In that case, excess photosynthate was converted to oil (Sanjaya *et al*., 2011). Carbohydrates produced from starch breakdown could also be a substrate for oil production in the A3OE lines. Sucrose is also one of the substrates for oil metabolism via the Kennedy pathway for TAG assembly, a key control of seed oil content (Chapman *et al.*, 2013). The high levels of sucrose in the final phase of grain development could trigger reallocation of the available carbohydrates into oil production.

This work has described for the first time the effect of increasing starch degradation during grain maturation resulting in release of soluble sugars and redirection to oil synthesis. We propose that starch turnover results in increased sucrose, which triggers enhanced fatty acid synthesis and TAG assembly.

Although the details of the manner in which sucrose level is sensed *in vivo* and how the over-expression of TaAMY3 in developing endosperm translates into the phenotypes described here remain to be fully understood, this work points to hitherto unknown regulatory controls of the major energy storage forms in grain development of one of the world’s three major cereal crops. This discovery could hold important implications for grain quality in wheat and the potential for translation of the mechanism to crop improvement in other species.

## Supplementary Material

Supplementary Data
