# Supplementary material for: Engineering α-amylase levels in wheat grain suggests a highly sophisticated level of carbohydrate regulation during development
Source: J Exp Bot. 2014 Jul 22;65(18):5443–57. doi: 10.1093/jxb/eru299 (PMC4157717; doi:10.1093/jxb/eru299)
Supplement: Supplementary Data [file supp_65_18_5443__index.html]

Engineering α-amylase levels in wheat grain suggests a highly sophisticated level of carbohydrate regulation during development — Engineering α-amylase levels in wheat grain suggests a highly sophisticated level of carbohydrate regulation during development — Supplementary Data 

# Engineering α-amylase levels in wheat grain suggests a highly sophisticated level of carbohydrate regulation during development

## Supplementary Data

Data files

**Files in this Data Supplement:**

- Supplementary Data - Supplementary Data
