# Supplementary material for: Engineering α-amylase levels in wheat grain suggests a highly sophisticated level of carbohydrate regulation during development
Source: J Exp Bot. 2014 Jul 22;65(18):5443–57. doi: 10.1093/jxb/eru299 (PMC4157717; doi:10.1093/jxb/eru299)
Supplement: Supplementary Data [file supp_eru299_jexbot122549_file001.pdf]

A

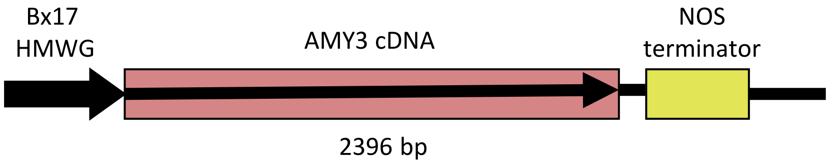

B

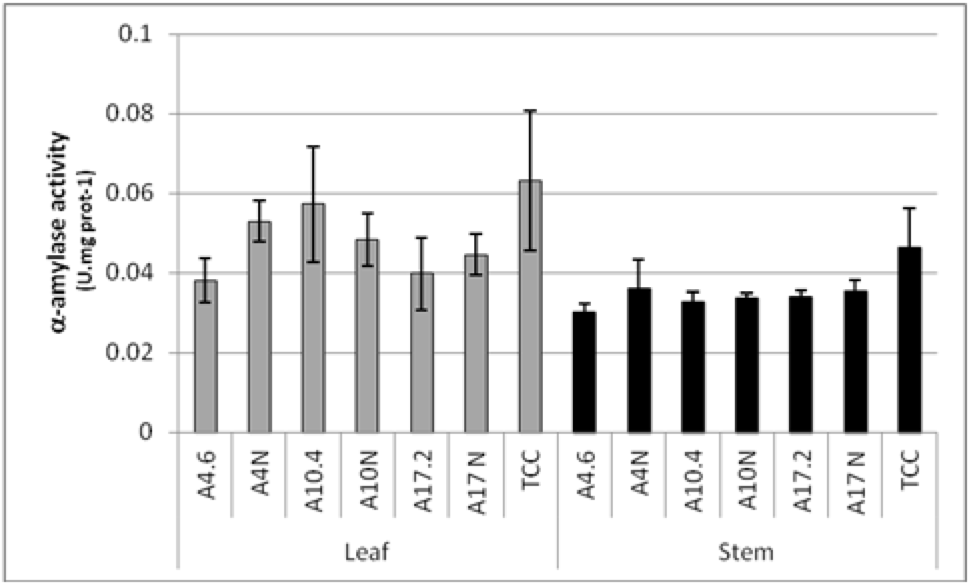

Figure S1

(A) Schematic representation of the pAMY3OEBx17 construct for wheat transformation. Bx17HMWG is the high molecular weight glutenin promoter. AMY3 cDNA is the sense from a cDNA for wheat AMY3. NOS terminator is from the nopaline synthase gene.

(B) α-amylase activity in Leaf (grey bar) and Stem (Black bar) tissues collected at 20 DPA from T3 plants of a representative panel of three homozygous lines from independent events, labelled A4.6, A10 .4 and A17.2, together with their isogenic negative segregants (A4N, A10N and A17N respectively) and tissue culture control (TCC). Activity expressed in Ceralpha units per mg protein (Triplicate measurements). Error bars show standard error values.

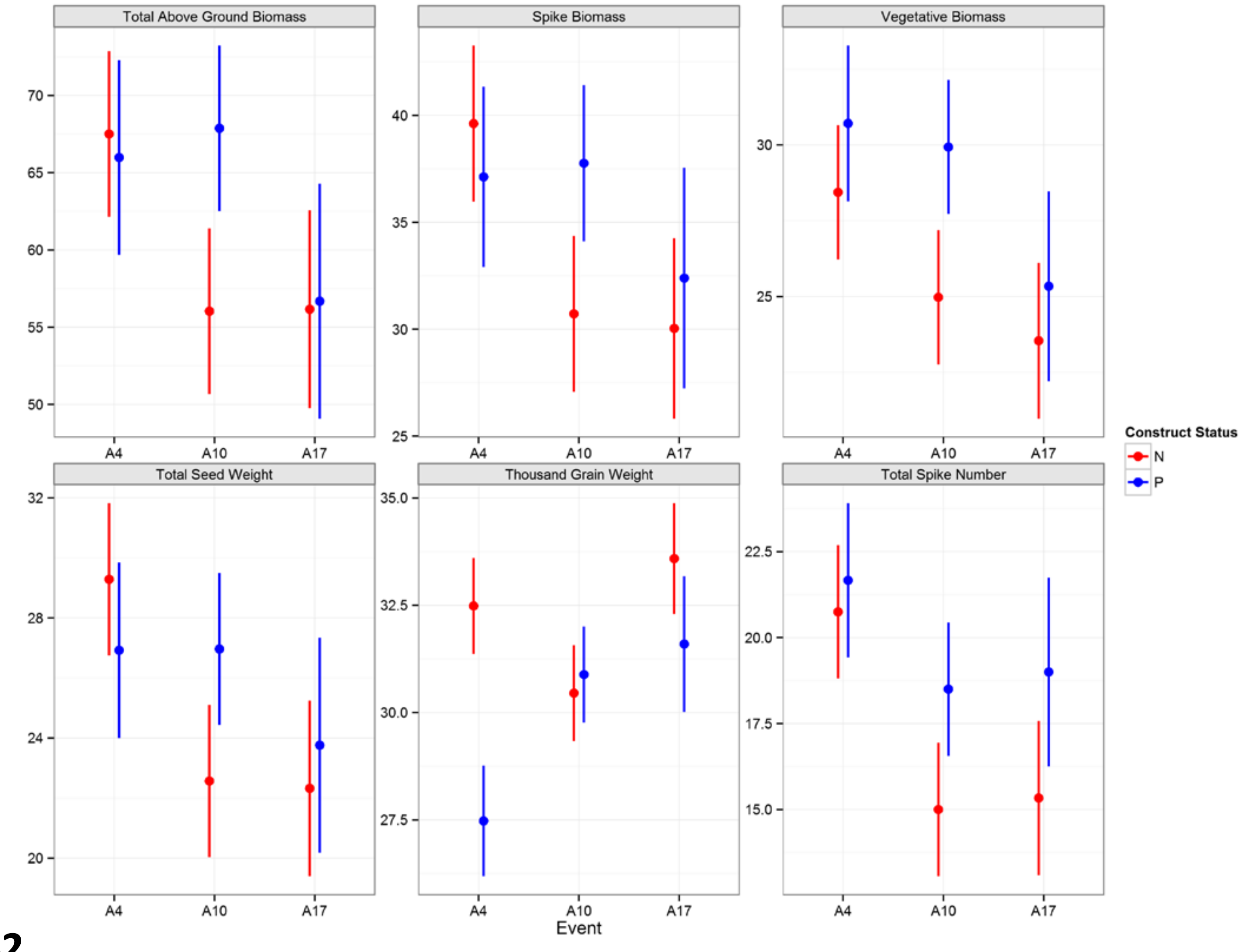

Figure S2

Morphological and phenotypical characterisation of Amylase 3 Overexpression lines in T3 plants. Positive AOE lines (Blue) are compared by pair to their isogenic negative segregants (Red). All figures are expressed in grams with the exception of the Total Spike number (count). Error bars show standard error values.

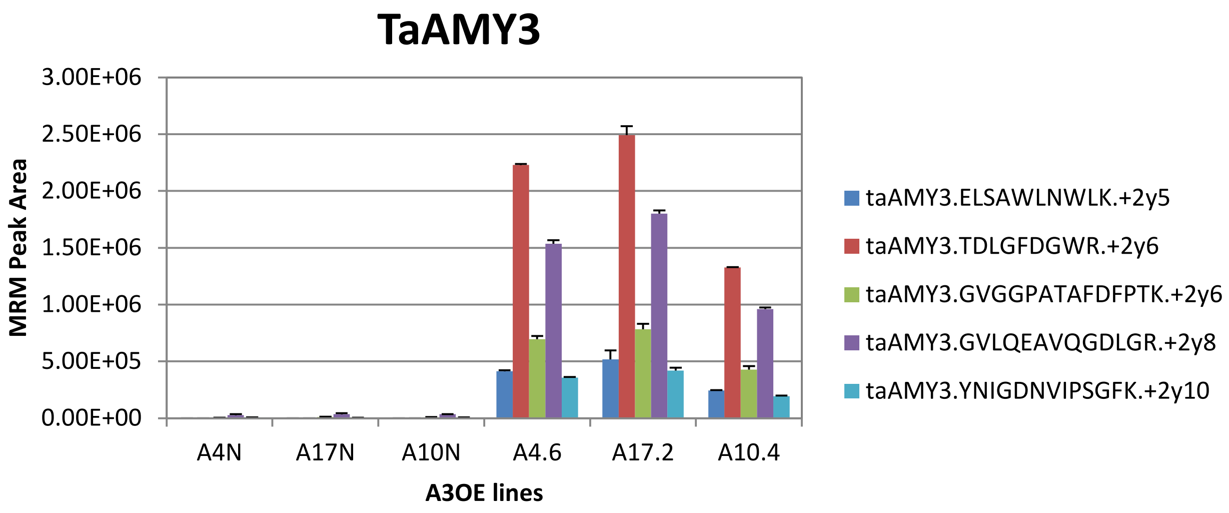

Figure S3

Relative quantification and comparison of TaAMY3 peptides using Mass Spectrometry. Proteins were extracted from mature grain of A3OE lines (A4.6, A10.4 and A17.2) and their isogenic negative controls (A4N, A10N and A17N) . Produced from trypsic digestion were identified and quantified using Mass spectrometry. Figure shows the relative peak area for each identified peptides. Peptide sequences are showing on the right hand side of the figure.

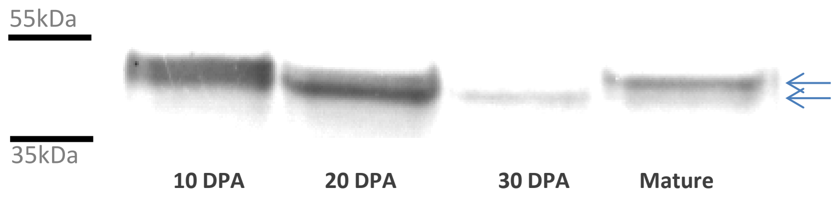

Figure S4

Representative Western-blot of A3OE line A10 using AMY3 specific antibody. Starch protein extracts were extracted from grain at 10, 20, 30 Days after anthesis (DPA) and at maturity. Two bands were detected across the grain development and indicated by the arrows. The 2 bands are present in similar proportion at 10 DPA. The smaller form is present in majority at 20 and 30 DPA while the larger band is present in majority in mature grain.

Event	Relative protein concentration increase (%)			Relative activity increase (%)		
	**Average**	**High**	**low**	**Average**	**High**	**low**
**A4**	16.6	53.1	1.0	81.8	392.3	0.2
**A8**	0.6	0.8	0.4	1.3	2.8	0.3
**A10**	4.4	7.9	2.1	8.6	33.3	0.4
**A11**	9.4	27.3	0.5	354.6	2295.5	0.5
**A12**	1.8	3.0	0.7	6.0	28.2	0.7
**A15**	1.1	1.9	0.5	1.4	3.1	0.2
**A16**	1.4	1.9	0.5	1.7	6.8	0.1
**A17**	10.9	25.2	2.2	37.1	149.0	0.6
**A18**	1.4	2.1	0.9	10.4	24.3	2.3
**A23**	2.8	4.6	0.3	77.9	183.2	0.3
**A24**	20.8	35.3	2.5	27.9	63.2	2.1
**A29**	1.5	2.6	1.2	1.2	2.8	0.2
**A38**	13.6	29.6	1.0	33.0	190.7	0.5
**A39**	13.6	20.6	7.3	145.1	304.7	53.6
**A40**	2.7	5.3	1.2	21.3	41.7	7.8

Table S1

Analysis of α-amylase 3 (AtAMY3) protein quantification and enzyme activity in 180 individual transgenic T2 seeds, from 15 independent transformation events. **Relative protein quantification**, compared to parental line BW26. 20 μg of protein per lane were run on a 12% (w/v) poly-acrylamide gel and probed using anti-AMY3 polyclonal antibodies. Membranes were then scanned and band intensity compared to BW26 was measured using ImageJ software. **Relative alpha-amylase activity**, compared to parental line BW26. Alpha-amylase activity was measured using Ceralpha kit, Megazyme. Three technical replicates were performed. Selected lines for further characterisation are shaded.
